# Sacubitril/Valsartan Improves Functional Capacity and Reverses LV Remodeling in Obese Patients with Hypertrophic HFpEF: A Randomized Open-Label Study

**DOI:** 10.3390/jcm15114286

**Published:** 2026-06-01

**Authors:** Artem Ovchinnikov, Alexandra Potekhina, Anastasiya Shchendrygina, Olga Svirida, Maria Sobolevskaya, Zoya Blankova, Maria Glezer, Fail Ageev

**Affiliations:** 1 Laboratory of Myocardial Fibrosis and Heart Failure with Preserved Ejection Fraction, National Medical Research Center of Cardiology Named After Academician E.I. Chazov, Academician Chazov St., 15a, 121552 Moscow, Russia; potehina@gmail.com (A.P.); olgasvirida@yandex.ru (O.S.); msobolevskaya95@mail.ru (M.S.); blankovazoya@yandex.ru (Z.B.); 2 Department of Clinical Functional Diagnostics, Russian University of Medicine of the Ministry of Health of the Russian Federation, Dolgorukovskaya St., 4, 127006 Moscow, Russia; 3 Department of Inpatient Medicine No. 2, M. Sechenov First Moscow State Medical University, Trubetskaya St., 8-2, 119991 Moscow, Russia; a.shchendrygina@gmail.com; 4 Department of Cardiology, The Vladimirsky Moscow Regional Research Clinical Institute, Shchepkina Street, 61/2, 129110 Moscow, Russia; 287ast@mail.ru; 5 Out-Patient Department, National Medical Research Center of Cardiology Named After Academician E.I. Chazov, Academician Chazov St., 15a, 121552 Moscow, Russia; ftageev@gmail.com

**Keywords:** sacubitril/valsartan, left ventricular hypertrophy, obesity, diastolic dysfunction, natriuretic peptides, heart failure with preserved ejection fraction

## Abstract

**Background:** Heart failure with preserved ejection fraction (HFpEF) has multiple phenotypic manifestations with heterogeneous treatment responses. **Objective:** To evaluate the effect of sacubitril/valsartan (Sac/Val) on functional capacity and cardiac remodeling in overweight/obese HFpEF patients with concentric left ventricular hypertrophy (LVH). **Methods**: Sixty-one overweight/obese HFpEF patients (body mass index ≥ 25 kg/m^2^) with hypertensive LVH (LV mass index ≥ 115 g/m^2^ for men or ≥94 g/m^2^ for women) were randomized to Sac/Val (100–400 mg a day; *n* = 30) versus the usual care group (*n* = 31) for 6 months. Changes in six-minute walk test distance (6MWTD) were the primary outcomes. Secondary outcomes included changes in echocardiographic parameters of cardiac structure and function, and N-terminal pro-brain natriuretic peptide (NT-proBNP). **Results**: After 6 months of Sac/Val therapy, 6MWTD increased, and E/e′ ratio, LV mass index, LA volume index, and NT-proBNP levels decreased compared with the usual care group (*p* < 0.05 for all). **Conclusions**: In overweight/obese patients with HFpEF and LVH, Sac/Val significantly improved functional capacity and reduced LV mass and filling pressure compared with standard medical therapy.

## 1. Introduction

Heart failure with preserved ejection fraction (HFpEF) accounts for approximately half of all HF cases. Its prevalence is increasing primarily due to an aging population and a high incidence of arterial hypertension, obesity, diabetes mellitus (DM), and atrial fibrillation. The prognosis for HFpEF is nearly as poor as for heart failure with reduced ejection fraction. Nevertheless, the development of effective therapeutic interventions is constrained by considerable heterogeneity in phenotypic manifestations observed in HFpEF [1].

Natriuretic peptide (NP) deficiency is a defining feature of HFpEF, characterized by decreased NP blood levels and diminished NP bioavailability [2]. NP deficiency syndrome results primarily from a combination of reduced production and enhanced clearance of NPs. Specifically, NP production declines due to concentric LV remodeling or hypertrophy, a common structural cardiac alteration observed in HFpEF [3], which reduces diastolic wall stress. Normally, increased diastolic wall stress during elevated LV filling pressures stimulates NP release, but hypertrophic ventricular changes blunt this trigger. Additionally, visceral adipose tissue expansion, a prevalent feature among overweight or obese HFpEF patients, triggers the production of signaling molecules that increase neprilysin activity and the expression of C-receptors for NPs, involved in the degradation and clearance of NPs [4]. In addition, morbid obesity contributes to NP deficiency by increasing epicardial adipose tissue, which compresses the heart chambers and impedes ventricular filling. This, in turn, leads to reduced ventricular distension and, consequently, to decreased NP secretion [5,6]. Thus, obese HFpEF patients with LV concentric remodeling/hypertrophy often have lower NP levels than HFpEF patients with a normal body mass index (BMI) and/or normal LV geometry. In turn, low NP bioavailability leads to vascular and ventricular fibrosis and hypertrophy, impaired relaxation, increased cardiomyocyte stiffness, and fluid retention [7].

Sacubitril/valsartan (Sac/Val) therapy addresses NP deficiency by increasing NPs’ availability and leads to many benefits, such as increased urine output, improved relaxation, reduced inflammation, fibrosis, and hypertrophy, all of which are vital in HFpEF [8]. However, in the PARAGON-HF (Prospective Comparison of Angiotensin Receptor-Neprilysin Inhibitor With Angiotensin-Receptor Blockers Global Outcomes in Heart Failure With Preserved Ejection Fraction) trial, Sac/Val showed only a trend toward improved outcomes in HFpEF [9]. Notably, just 12% of trial patients had concentric LVH, and only half were obese [10]. So, many participants likely did not have significant NP deficiency, which may have limited Sac/Val’s effects. We hypothesized that among patients with HFpEF, those who are overweight or obese and have LVH—a combination associated with NP deficiency—would exhibit the greatest improvement in exercise capacity and cardiac structure and function in response to increased NP bioavailability from Sac/Val. This study specifically examines the impact of Sac/Val on these outcomes in this target population.

## 2. Materials and Methods

### 2.1. Study Design

This study was a 24-week, single-center, randomized, parallel-group, open-label trial. The study aimed to evaluate the effect of Sac/Val on functional capacity and echocardiographic parameters of LV remodeling in overweight/obese HFpEF patients. It consisted of treatment with either Sac/Val (100–400 mg a day) or ongoing therapy with angiotensin-converting enzyme inhibitors (ACEi) or angiotensin receptor blockers (ARB) for 6 months. It was an investigator-initiated, investigator-led trial conducted at the Institute of Clinical Cardiology. This study was approved by the Institutional Review Board of the Institute of Clinical Cardiology (Protocol No. 1/349, 1 February 2018) and complied with the Declaration of Helsinki. All patients provided written informed consent. The trial has been registered at ClinicalTrials.gov (Identifier: NCT03928158) and has been reported in accordance with the CONSORT statement.

### 2.2. Study Population

The study enrolled adults aged ≥ 40 years with stable HF, New York Heart Association (NYHA) functional class II-III, preserved LV EF (≥50%), concentric LVH, and evidence of elevated LV filling pressure, as verified by echocardiography at rest or during exercise [11]. In addition, all study participants were required to be overweight (body mass index 25.0–29.9 kg/m^2^) or obese (body mass index ≥ 30.0 kg/m^2^). Those patients with alternative causes of LVH, asymmetrical or eccentric LVH, secondary hypertension, artificial cardiac rhythm or left bundle branch block, significant left-sided structural valve disease, infiltrative or inflammatory myocardial diseases, diseases associated with isolated right ventricular (RV) failure, or noncardiac conditions precluding participation were excluded. As one of the study’s objectives was to evaluate cardiac reserves during exercise, patients with chronic atrial flutter/fibrillation were also excluded.

### 2.3. Study Procedures

Eligible participants were randomly assigned in an open-label manner to receive Sac/Val or to the usual care group (individualized renin–angiotensin–aldosterone system [RAAS]-based drug therapy [ACEi or ARB]) at a 1:1 ratio for 6 months. Sequentially numbered opaque sealed envelopes were used to conceal the allocation. An external co-worker opened the envelope after obtaining informed consent from each patient. Patients in the Sac/Val group received the drug at an initial dose of 24/26 mg twice daily if they had previously taken a low dose of RAAS blockers (e.g., enalapril < 10 mg twice daily or valsartan < 160 mg twice daily) or at an initial dose of 49/51 mg twice daily if they had previously taken high-dose RAAS blockers (e.g., enalapril 10 mg twice daily or valsartan 160 mg twice daily), with the Sac/Val dose increased over 4 weeks to a maximum tolerated dose of 97/103 mg twice daily. Patients in the usual care group continued to take the ACE inhibitors or ARB that had previously been prescribed to treat cardiovascular disease and other conditions typically associated with HFpEF, such as AH, DM, and coronary artery disease (CAD). This approach to organizing a usual care group has been previously used in the randomized clinical trial PARALLAX with Sac/Val in patients with HFpEF, aiming to reflect standard medical practice and guidelines for the treatment of these comorbidities [12]. If a patient in the usual care group was taking a low dose of RAAS blockers, the dose was up-titrated to a maximum tolerated dose (e.g., enalapril 10 mg twice daily or valsartan 160 mg twice daily).

The first dose of Sac/Val was administered no earlier than 36 h after discontinuing an ACE inhibitor or immediately after randomization if the patient was taking an ARB at the time of enrolment in the study. If a side effect developed while taking Sac/Val, study staff could recommend discontinuation or a return to a previously tolerated lower dose. Baseline cardiovascular medical therapy had remained unchanged for at least 3 months before randomization and throughout the follow-up period, except for increasing diuretic dose if dyspnoea worsened.

Baseline procedures included: the six-minute walk test distance (6MWTD), echocardiography at rest and during exercise, and blood sampling for biomarker assessment. Follow-up visits were conducted every 2 weeks during up-titration of the study drug and RAAS blockers, and subsequently every month until the end of follow-up. After 6 months, the baseline procedures were repeated. To minimize potential measurement bias, access to patient allocation was restricted to investigators performing key procedures, such as the 6MWTD, echocardiography, and blood tests.

### 2.4. Study Endpoints

The primary endpoint of this study was the change in 6MWTD from baseline to 6 months of Sac/Val therapy, compared with the usual care group. The secondary objectives were changes in the E/e′ ratio and PASP at rest and during exercise, as well as the LA volume index, LV mass index, and biomarkers, after 6 months of therapy.

### 2.5. Echocardiography

Echocardiographic assessment was performed using a Vivid E95 ultrasound system (GE Healthcare, Horton, Norway). The wall thickness, chamber dimensions/volumes, and LV EF were determined in accordance with the current guidelines [13]. To calculate LV mass, we used the 2D-guided M-mode method. This method relies on linear measurements of the LV diastolic diameter and wall thickness, along with geometric formulas, to calculate the LV myocardial volume [13]. The measured LV mass values were indexed to body surface area. LVH was defined as an LV mass index greater than 115 g/m^2^ in men and greater than 95 g/m^2^ in women. LVH was classified as mild when the LV mass index was between 116 and 131 g/m^2^ in men and between 96 and 108 g/m^2^ in women; moderate when the LV mass index was between 132 and 148 g/m^2^ in men and between 109 and 121 g/m^2^ in women; and severe when the LV mass index was greater than 148 g/m^2^ in men and greater than 121 g/m^2^ in women. Relative wall thickness (RWT) was defined as septal thickness plus posterior wall thickness divided by LV end-diastolic dimension. Concentric hypertrophy was diagnosed based on an increase in LV mass index and an RWT > 0.42.

LV diastolic function was assessed by measuring the following: transmitral inflow peak early diastolic and late diastolic velocities (E and A, respectively) and their ratio (E/A), the averaged mitral annulus relaxation velocity (e′ velocity), E/e′ ratio, LV volume index, and the peak tricuspid regurgitation (TR) velocity. The severity of LV diastolic dysfunction (DD) was determined according to the 2016 American Society of Echocardiography and the European Association of Cardiovascular Imaging criteria [11]. In short, an E/A ratio of 0.8 or less and a peak E-wave velocity of 50 cm/s or less indicate grade I LVDD. Conversely, an E/A ratio of 2 or greater suggests grade III LVDD. For patients between these two extremes, determine how many of the high LV filling pressure criteria are met: E/e′ > 14, LAVI > 34 mL/m^2^, and peak TR velocity > 2.8 m/s. If two or more of these criteria are met, then grade II LVDD is present. If none of these criteria are met, then grade I LVDD is present [11]. Elevated LV filling pressure at rest was confirmed by the detection of grade II–III LVDD, and during supine cycling by the observation of exercise-induced increases in average E/e′ > 14 and TR velocity > 2.8 m/s [11]. The pulmonary artery systolic pressure was calculated by adding the peak TR velocity to the estimated right atrial (RA) pressure, which was determined by the size and collapse of the inferior vena cava.

Deformation analysis was performed using two-dimensional echocardiography with speckle tracking and a dedicated software package (Echo-Pac version 203, GE Healthcare) at 50–80 frames per second. The LA endocardial boundaries were defined automatically using software. If necessary, the sonographer could adjust the measurements based on the visualized endocardial boundaries. LA strain was calculated as the average peak positive strain value during ventricular systole in six segments of the LA in a nonforeshortened apical four-chamber view to calculate reservoir (LASr) strain [14]. An abnormal LASR was defined as less than 23% [15].

LV diastolic SR during isovolumic relaxation (SR_IVR_) was determined as the average value of the longitudinal positive strain rate peak during the LV isovolumic relaxation period from all LV segments in the apical 4-chamber view [16]. The E/SR_IVR_ ratio was used as a measure of LV filling pressure.

All echocardiographic measures at rest represent an average of at least three beats.

### 2.6. Diastolic Stress Test (DST)

All subjects performed an incremental stepwise test on a supine cycle ergometer (ERG 911 BP/LS, Shiller, Baar, Switzerland) at 60 rpm. The test started with 3 min at a low workload of 25 W, followed by 25 W increments every 3 min until the patient reached severe chest pain and/or a diagnostic ST-segment shift, reached the maximal predicted heart rate, or developed limiting symptoms. During the exercise, heart rate and blood pressure were continuously monitored. During the test, the changes in LV filling pressures (the E/e′ ratio and TR velocity), as well as some cardiac reserves, such as LV diastolic (changes in e′ velocity from rest to maximal exercise), and LV preload (changes in E velocity from rest to maximal exercise), were analyzed. Elevated LV filling pressure at exercise was verified if exercise-induced elevations in E/e′ ratio (average E/e′ ratio > 14) and TR velocity (>2.8 m/s) were observed [11].

### 2.7. Biomarker Assessment

Blood levels of biomarkers of myocardial stress (N-terminal pro-brain natriuretic peptide [NT-proBNP]), inflammation (high-sensitivity C-reactive protein [hsCRP]), and extracellular matrix homeostasis (osteopontin) were analyzed. Blood samples were collected into tubes without an anticoagulant (to obtain serum) or with citrate anticoagulant (to obtain plasma) via a venous puncture after a 20-min supine resting period. The samples were centrifuged immediately at 2000× *g* for 10 min and stored below 80 °C. NT-proBNP was assayed in the plasma, whereas osteopontin and hsCRP were assayed in the serum. NT-proBNP levels were measured using an automated electrochemiluminescence immunoassay (Roche Diagnostics, Mannheim, Germany). hsCRP concentration was measured by laser microscale nephelometry using a BN ProSpec laser light-scattering system (Behring, Marburg, Germany). An ELISA kit for osteopontin (Quantikinew Human Osteopontin (OPN) Immunoassay, R&D Systems Inc., Minneapolis, MN, USA) was used.

### 2.8. Statistical Analysis

The change in 6MWTD, the primary endpoint, was used to estimate the sample size required to achieve adequate statistical power for the current study. Based on our preliminary data, we deemed the average difference of at least 18 m between the Sac/Val group and the usual care group at six months of therapy from baseline to be significant. Based on our previous study, we expected the standard deviation of the differences to be 24 m [17]. For α = 0.05 (two-tailed), we determined that a sample size of 29 patients in each group would be required to achieve 80% power.

Normally distributed data are presented as the means ± standard deviations; nonnormally distributed data are presented as medians and interquartile ranges; categorical variables are reported as numbers and percentages of observations. For normally distributed variables, one-way analysis of variance was used to assess changes from baseline; for nonnormally distributed variables, the Wilcoxon test was used. The differences in parameters at baseline and after treatment between the Val/Sac and the usual care groups were tested via Student’s *t* test for normally distributed variables and the Mann–Whitney U test for nonnormally distributed variables. The treatment effects are presented via point estimates and 95% confidence intervals (CIs). Partial correlation coefficients, adjusted for age and sex, were calculated to assess the relationships between continuously distributed variables. A *p* value of <0.05 was considered statistically significant. Statistical analysis was performed using standard software (MedCalc, version 19.5.3, Ostend, Belgium).

## 3. Results

**Figure 1:**
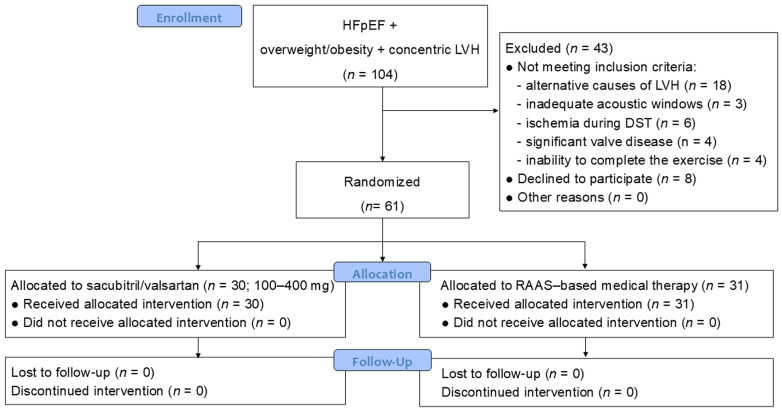
Flow chart of patient enrolment. DST, diastolic stress test; HFpEF, heart failure with preserved ejection fraction; LVH, left ventricular hypertrophy; RAAS, renin–angiotensin–aldosterone system.

Patient enrollment and the follow-up period lasted from June 2019 to June 2023. During this time, 104 patients with HFpEF, overweight/obesity, and concentric LVH were screened. Thirty-five patients did not meet the inclusion/exclusion criteria (mainly due to alternative causes of LVH), and eight patients refused to participate in the active phase of the study. Thus, the final cohort comprised 61 participants (Figure 1).

### 3.1. Patient Baseline Characteristics

Sixty-one participants were included in the final cohort; 30 received Sac/Val, and 31 received previously prescribed RAAS therapy (the usual care group; Figure 1).

The mean age of patients was 65.6 ± 8.5 years, and 57% were women. All patients were obese/overweight with multiple comorbidities (Table 1): paroxysmal atrial fibrillation (40%), chronic kidney disease (39%), DM (34%), and CAD (31%). At the time of inclusion in the study, all patients were taking RAAS blockers; about 70% received beta-blockers, statins, and loop diuretics, 30% were on mineralocorticoid receptor antagonists. Most study participants were enrolled before the era of modern HFpEF therapy; only a small number were taking sodium-glucose cotransporter 2 inhibitors, mainly because of DM (Table 1).

Seventy per cent had increased LV filling pressure at rest (grade II-III DD); the remaining patients had normal LV filling pressure at rest (grade I DD) but LV filling pressure elevation during DST. Despite the high proportion of patients presenting with severe LVDD and elevated resting filling pressure, the median NT-proBNP level was only mildly elevated (217 pg/mL). Moreover, 25% of patients had normal NT-proBNP levels (<125 pg/mL).

The study groups were comparable in their demographic and hemodynamic characteristics, as well as in their current medications (Table 1).

### 3.2. Study Treatment

No patients in either group were lost to follow-up. By the end of the study, 19 patients (63%) in the Sac/Val group were taking the target dose of 400 mg per day, while the remaining 11 patients were taking 100–200 mg per day. In the usual care group, 27 of 31 (87%) received ARB, and the remaining 4 were taking ACEi.

An increase in diuretic dosage was required for one patient in the Sac/Val group and two patients in the usual care group due to worsening dyspnoea. Both groups demonstrated comparable reductions in blood pressure, with the Sac/Val group showing a significant intragroup decrease in both systolic and diastolic BP compared with baseline (*p* < 0.05 for both; Table 2). Seven patients in the Sac/Val group developed asymptomatic arterial hypotension (systolic BP < 100 mm Hg), requiring a reduction in the dose or discontinuation of up-titration; no cases of arterial hypotension occurred in the usual care group (*p* = 0.005 for the difference between groups). No cases of angioedema, hyperkalemia (potassium ≥ 5.5 mmol/L), or worsening renal function (worsening of the eGFR by ≥25% from baseline) were observed in either group.

### 3.3. Functional Capacity

**Figure 2:**
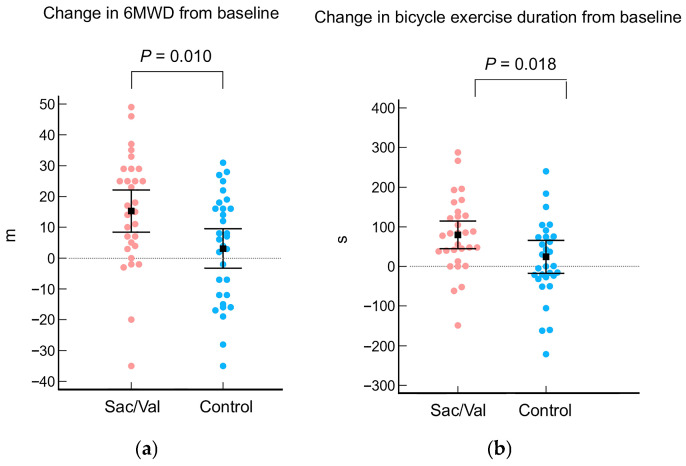
(**a**) Individual and mean changes from baseline (95% CI) in the 6-min walk distance, and (**b**) bicycle exercise duration in both study groups. Circles represent individual changes, the squares indicate the means, and the error bars indicate the 95% CIs. Sac/Val, sacubitril/valsartan; 6MWDT, 6-min walk test distance.

After 6 months of therapy, 6MWTD increased by 15 (95% CI, 8 to 22) m in the Sac/Val group, compared to an increase of only 3 (95% CI, −3 to 10) m in the usual care group (*p* = 0.010 for between-group differences; Figure 2a). In the Sac/Val group, a significantly higher proportion of patients achieved a prominent increase in the 6MWTD (≥15 m) than did the usual care group (*n* = 16 [53%] vs. *n* = 6 [19%], respectively; *p* = 0.006). Moreover, a smaller proportion of patients had a ≥15 m reduction in the 6MWTD (*n* = 2 [7%] vs. *n* = 7 [23%], respectively; *p* = 0.08).

After 6 months, the exercise duration during the bicycle test increased by 80 s (95% CI, 44 to 111) s in the Sac/Val group and only by 22 s (95% CI, −16 to 59) s in the usual care group (*p* = 0.02 for between-group differences; Figure 2b).

### 3.4. Resting Echocardiographic Parameters

**Figure 3:**
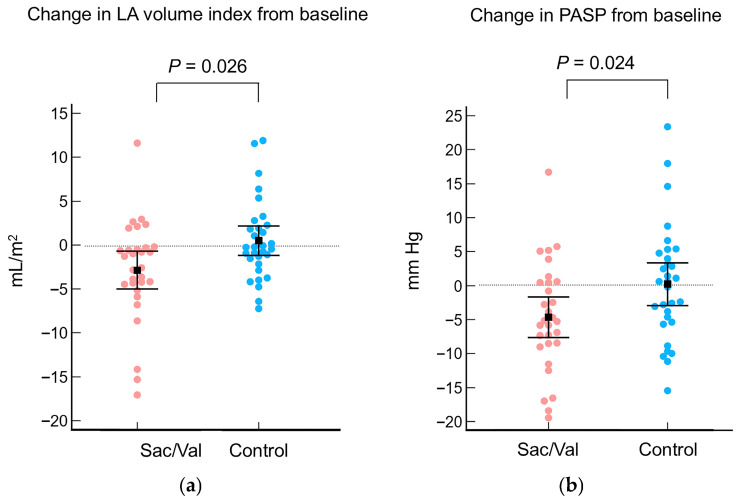

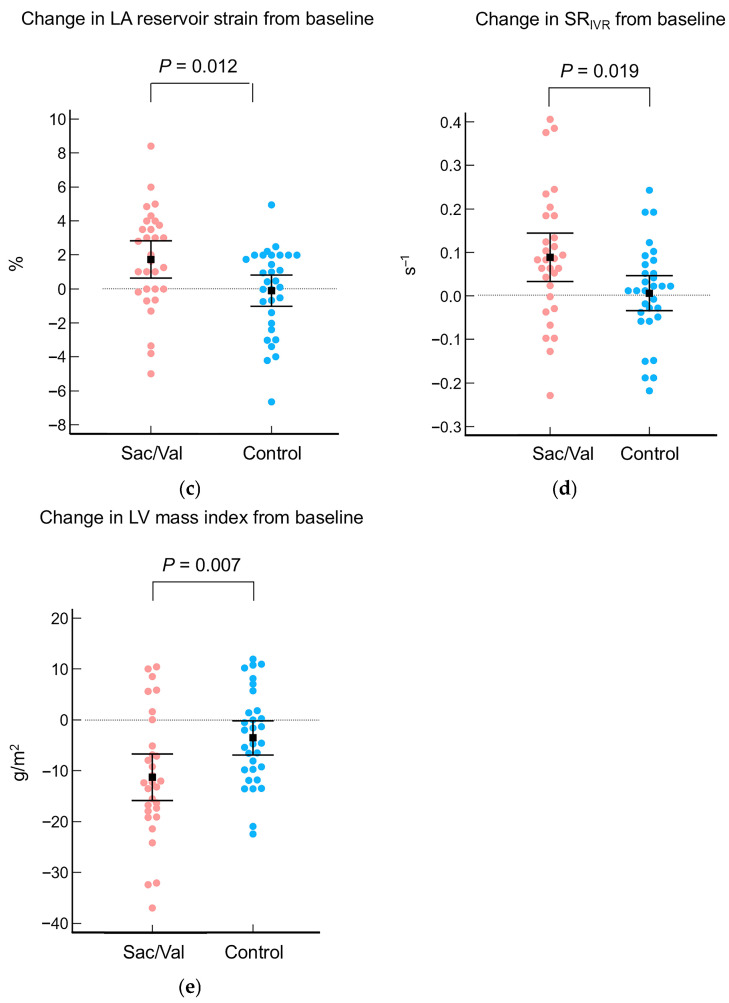
(**a**) Individual and mean changes from baseline (95% CI) in the LA volume index, (**b**) PASP, (**c**) LA reservoir strain, (**d**) SR_IVR_, and (**e**) LV mass index in both study groups. Circles represent individual changes, the squares indicate the means, and the error bars indicate the 95% CIs. LA, left atrial; LV, left ventricular; PASP, pulmonary artery systolic pressure; RVOT, right ventricular outflow tract; Sac/Val, sacubitril/valsartan; SR_IVR_, global strain rate during the isovolumic relaxation period.

After 6 months of therapy, the Sac/Val group demonstrated improvements in all echocardiographic parameters associated with LV filling pressure. Thus, the mean change in the E/e′ ratio was −1.1 (95% CI, −2.0 to −0.1); the E/SRIVR ratio was −110 (95% CI, −166 to −54) cm, the LA volume index was −2.4 (95% CI, −4.2 to −0.7) mL/m^2^; and estimated PASP was −4.9 (95% CI, −7.6 to −1.8) mm Hg (for all *p* < 0.05 compared with baseline, Figure 3a,b). These parameters did not change in the usual care group, resulting in significant between-group differences (all *p* < 0.05).

In the Sac/Val group, the reduction in LV filling pressure was accompanied by a significant improvement in the left heart chamber functional parameters, namely an increase in LA longitudinal strain in the reservoir phase (LASr) and an increase in SR_IVR_ (reflecting improved LV active relaxation; for both *p* < 0.05 compared to baseline). By contrast, there was no change in these parameters in the comparison group, resulting in significant between-group differences (*p* < 0.05 for both; Figure 3c,d).

A mild but significant correlation was found between changes in the 6MWTD, study primary endpoint, and the dynamics of variables associated with LV filling pressure (LAVI: r = −0.33; *p* < 0.01, LVMI: r = −0.27; *p* = 0.04, LASr: r = 0.27; *p* = 0.04, PASP: r = −0.29; *p* = 0.02) during follow-up, underscoring the importance of reducing LV filling pressure for functional improvement in patients with HFpEF.

After 6 months, the LV mass index in the Sac/Val group decreased significantly by 12 (95% CI, −16 to −7 g/m^2^) g/m^2^, while in the usual care group it decreased by only 4 (95% CI, −7 to 0) g/m^2^, resulting in a significant between-groups difference (*p* = 0.007, Table 2, Figure 3e). Changes in LV mass index during the follow-up correlated with dynamics of LV filling pressure-associated parameters, such as the E/e′ ratio (r = 0.31, *p* = 0.02), the E/SR_IVR_ ratio (r = 0.48, *p* < 0.001), and PASP (r = 0.26, *p* = 0.04). These correlations emphasize the impact of LVH regression on the normalization of LV filling pressure.

The study found no differences between the groups in the effects on LV EF and volumes over time.

In the Sac/Val group, changes in systolic blood pressure correlated poorly with changes in key echocardiographic parameters, such as LV mass index (r = 0.13, *p* = 0.20), E/e′ ratio (r = 0.14, *p* = 0.14), and LA volume index (r = 0.09, *p* = 0.63), and PASP (r = 0.31, *p* = 0.09).

### 3.5. Cardiac Reserves

**Figure 4:**
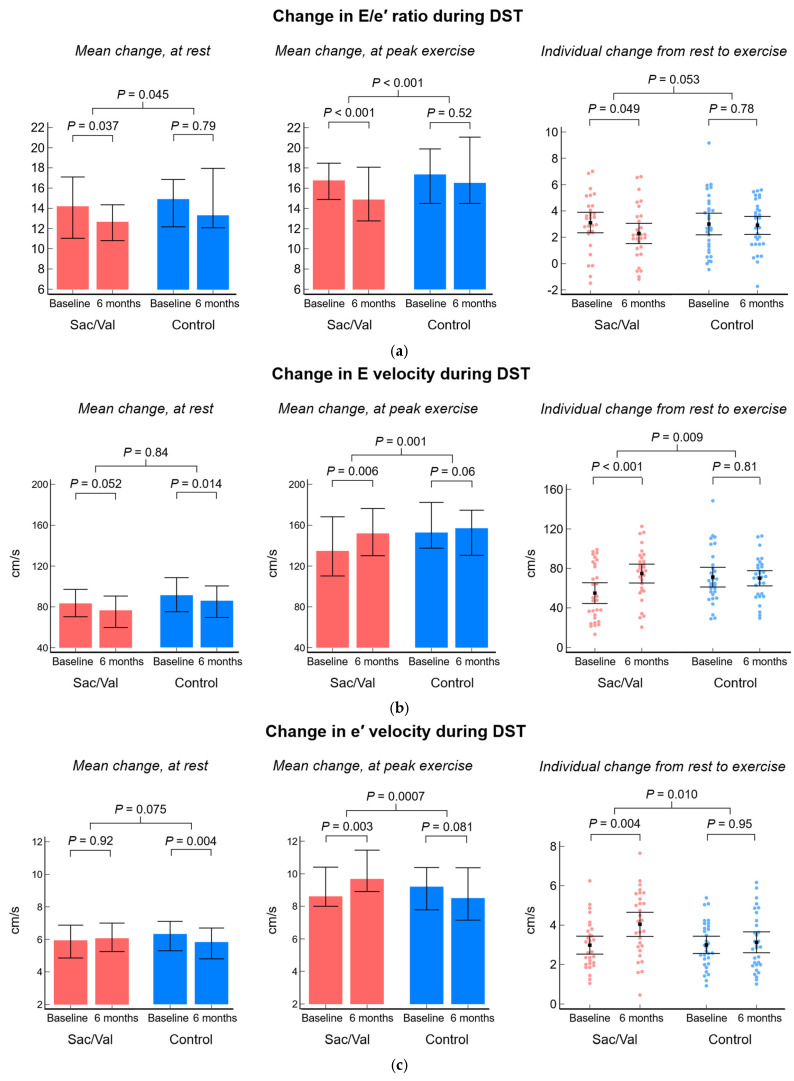
(**a**) The mean and individual changes in the E/e′ ratio, (**b**) E velocity, and (**c**) e′ velocity at rest and during cycle exercise at baseline and after 6 months in both study groups. The bars indicate the medians, the squares indicate the means, the markers (error bars) indicate the interquartile ranges or 95% confidence intervals, and circles indicate the individual changes. DST, diastolic stress test; E, early inflow velocity; e′, averaged annulus relaxation velocity; Sac/Val, sacubitril/valsartan.

After 6 months, both the E/e′ ratio and TR velocity significantly decreased not only at rest (see above for details), but also at the peak of exercise (for both *p* < 0.01 vs. baseline). In contrast, no significant changes were observed in the usual care group (for both *p* < 0.05 for between-group differences, Table 3). The study groups exhibited distinct patterns in the dynamics of the E/e′ ratio during exercise. In the Sac/Val group, the E/e′ ratio increment during exercise significantly decreased (from 3.2 at baseline to 2.2 at the end of the study, *p* = 0.049). In contrast, in the usual care group, the E/e′ ratio increment during exercise increased slightly from 2.9 to 3.0 (*p* = 0.78), resulting in nearly significant between-group differences (*p* = 0.053, Table 3, Figure 4a).

The reduction in LV filling pressure observed in the Sac/Val group was accompanied by improvements in LV diastolic and preload reserves (a greater increase in e′ velocity and E velocity during exercise, respectively, for both *p* < 0.01 vs. baseline). These reserves did not change significantly in the usual care group, resulting in significant between-group differences (for both, *p* < 0.05; Table 3, Figure 4b,c). Interestingly, in the Sac/Val group, LV preload (as assessed by E velocity) decreased at rest (*p* = 0.052 vs. baseline) but increased at peak exercise (*p* < 0.01 vs. baseline), resulting in a substantial increase in preload reserve.

After 6 months, there was a tendency toward a greater increase in TAPSE during exercise (which reflects an increase in RV contractile reserve) in the Sac/Val group than in the usual care group (*p* = 0.089 for between-group differences; Table 3). This led to a significantly higher TAPSE at peak exercise in the Sac/Val group than in the usual care group (*p* = 0.027, Table 3). A significant correlation was found between changes in LV preload reserve (changes in E velocity increase during exercise) and RV contractile dynamics (changes in TAPSE increase during exercise) in the Sac/Val group: r = 0.40, *p* = 0.03. This association suggests that restoring the contractile reserve of the RV during Sac/Val therapy plays an important role in improving LV filling during exercise, thereby restoring LV preload reserve.

### 3.6. Biomarkers

**Figure 5:**
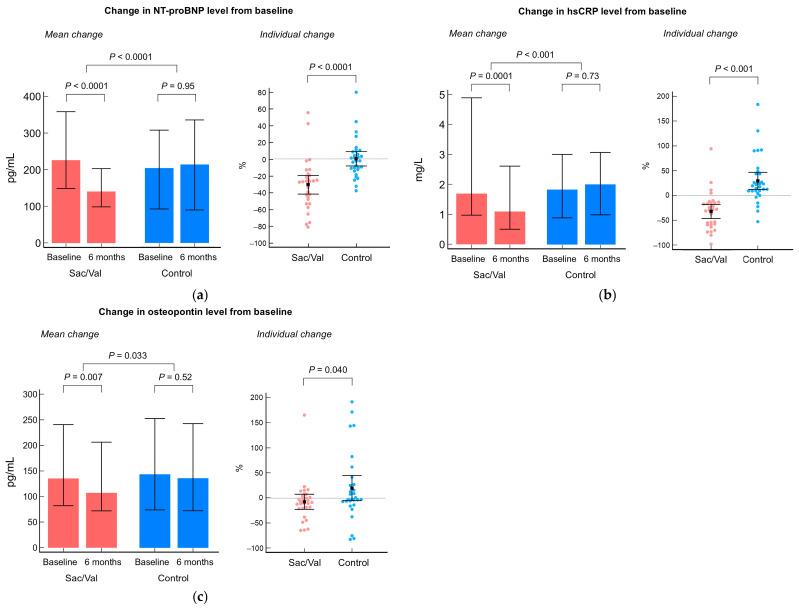
(**a**) The mean and individual changes from baseline in NT-proBNP, (**b**) hsCRP, and (**c**) osteopontin blood levels in both study groups. The bars indicate the medians, the squares indicate the means, the markers (error bars) indicate the interquartile ranges or 95% confidence intervals, and circles indicate the individual changes. hsCRP, high-sensitivity C-reactive protein; NT-proBNP, N-terminal pro-brain natriuretic peptide; Sac/Val, sacubitril/valsartan.

After 6 months, the median NT-proBNP blood level decreased by 81 (95% CI, −143 to −54) pg/mL in the Sac/Val group. Still, it increased by 1 pg/mL (95% CI, −15 to 29) in the usual care group, resulting in a significant between-group difference of 31% (95% CI, 20 to 44%; *p* < 0.001; Table 2, Figure 5a). Notably, NT-proBNP levels decreased during the study in all but 2 patients treated with Sac/Val, with half (15 of 30) experiencing a decrease of more than 30% (Figure 5a). Over the 6-month observation period, the change in NT-proBNP levels observed among all study participants correlated with the dynamics of 6MWTD (r = −0.35, *p* < 0.01), as well as with the dynamics of several echocardiographic parameters associated with LV filling pressure, including the E/e′ ratio at rest (r = 0.44, *p* < 0.001) and at peak exercise (r = 0.35, *p* = 0.006), E/SR_IVR_ ratio (r = −0.35, *p* = 0.005), LASr (r = −0.31, *p* = 0.014), and PASP (r = 0.26, *p* = 0.045).

Significant decreases in hsCRP and osteopontin blood levels were observed in the Sac/Val group compared with the usual care group (*p* < 0.001 and *p* = 0.033, respectively; Table 3, Figure 5b,c). The changes in hsCRP levels during study treatment correlated with the dynamics of 6MWTD (r = −0.42, *p* < 0.001), as well as with the changes in echocardiographic parameters: E/e′ ratio at peak exercise (r = 0.43, *p* < 0.001), LVMI (r = 0.30, *p* = 0.018), and e′ velocity at peak exercise (r = −0.39, *p* = 0.002). Additionally, a correlation was observed between changes in hsCRP and NT-proBNP at 6 months (r = 0.44, *p* < 0.001). All these relationships may indicate that the anti-inflammatory properties of RAAS blockers contribute to reducing elevated LV filling pressure—the main cause of exercise intolerance and the trigger of BNP production in HFpEF patients.

### 3.7. Comparison of Different Doses of Sac/Val

**Figure 6:**
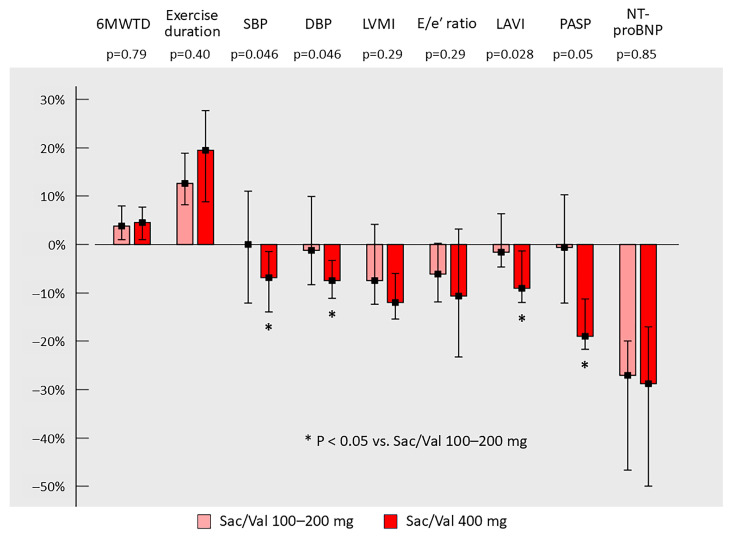
The effect of different doses of Sac/Val on key clinical and echocardiographic parameters in HFpEF patients with overweight/obesity and LV hypertrophy. The data are presented as percentage changes from baseline. Bars and squares indicate mean changes, while markers (error bars) indicate 95% confidence intervals. DBP, diastolic blood pressure; E, early inflow velocity; e′, averaged annulus relaxation velocity; LAVI, left atrial volume index; LVMI, left ventricular mass index; NT-proBNP, N-terminal pro-brain natriuretic peptide; PASP, pulmonary artery systolic pressure; Sac/Val, sacubitril/valsartan; SBP, systolic blood pressure; 6MWTD, 6-min walk test distance.

As quite a large proportion of patients (37%) in the Sac/Val group took the drug at a non-target dose (<97/103 mg BID), we were able to compare the effect of different Sac/Val doses (target dose of 97/103 mg BID vs. non-target dose) on key clinical and hemodynamic parameters (study endpoints). Sac/Val at the target dose outperformed Sac/Val at a lower dose in its effect on systolic and diastolic blood pressure, as expected. In addition, the target dose of Sac/Val also had a more significant effect on two key echocardiographic parameters associated with LV filling pressure: the LV volume index and PASP (Figure 6). Although there were no significant differences between different doses of Sac/Val in the changes in 6MWTD, exercise duration during the bicycle test, mitral E/e′ ratio, LVMI, and NT-proBNP, improvements in these parameters numerically were higher in individuals receiving the target dose of Sac/Val than in those receiving a lower dose (Figure 6).

### 3.8. The Effects of Sac/Val Depend on the Severity of LVH

**Figure 7:**
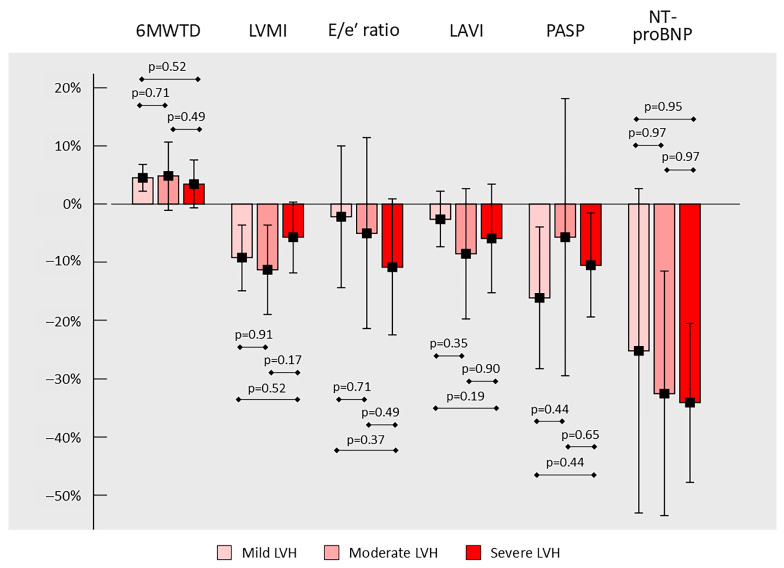
The effect of Sac/Val on key clinical and echocardiographic parameters in HFpEF patients depending on the severity of LVH. The data are presented as percentage changes from baseline. Bars and squares indicate mean changes, while markers (error bars) indicate 95% confidence intervals. E, early inflow velocity; e′, averaged annulus relaxation velocity; LAVI, left atrial volume index; LVH, left ventricular hypertrophy; LVMI, left ventricular mass index; NT-proBNP, N-terminal pro-brain natriuretic peptide; PASP, pulmonary artery systolic pressure; Sac/Val, sacubitril/valsartan; 6MWTD, 6-min walk test distance.

Given that advanced LVH carries a risk of irreversible myocardial changes that may compromise treatment efficacy, we analyzed the extent to which the clinical and hemodynamic effects of Sac/Val depend on LVH severity. This analysis demonstrated a comparable effect of Sac/Val on key parameters, irrespective of LVH severity (Figure 7).

## 4. Discussion

In this randomized, open study of overweight/obese HFpEF patients with concentric LVH, who are prone to exhibiting the NP deficiency syndrome, treatment with Sac/Val significantly improved exercise capacity compared with conventional RAAS inhibition-based medical therapy. This clinical benefit appeared to result from reductions in LV mass and filling pressure (at rest and during exercise). Therefore, our data support the use of Sac/Val in these selected HFpEF patients, particularly given their distinct pathophysiological profile.

The role of therapy in alleviating symptoms and slowing the progression of HF in patients with NP deficiency syndrome remains unclear, as such patients are typically excluded from HFpEF studies due to low NT-proBNP levels. However, neprilysin inhibition may provide therapeutic benefits by producing a direct natriuretic effect and reducing circulating aldosterone, since potentiated NPs inhibit aldosterone secretion and counteract its effects [18,19]. Enhancing the effects of natriuretic peptides has been shown to cause significant lipolytic and anti-inflammatory responses, inhibit cardiac fibrosis, and promote capillary vasculogenesis [20,21,22,23]. Therefore, increasing NP bioavailability via neprilysin inhibition may help alleviate plasma volume expansion, cardiac fibrosis, and microcirculatory rarefaction seen in obesity-related HFpEF, and holds promise for reducing myocardial stiffness, lowering LV filling pressure, and improving exercise tolerance.

Given that obesity and concentric LV remodeling reduce NPs’ bioavailability and their beneficial effects, we assumed that increasing NPs’ bioavailability by inhibiting neprilysin in these HFpEF individuals could be highly beneficial. Our study demonstrated the relevance of this assumption, with significant improvements observed in all key research domains—clinical (functional capacity), hemodynamic (LV filling pressure), morphological (LV mass), and biological (NT-proBNP and biomarkers of inflammation and fibrosis). Nevertheless, the PARAGON-HF trial, which involved 4822 patients with HF and an EF ≥ 45%, found that Sac/Val therapy did not significantly reduce the primary composite outcome of cardiovascular death or HF hospitalization compared with valsartan therapy (relative risk reduction of 13%; *p* = 0.06). Notably, PARAGON-HF participants had much lower rates of concentric LVH (12%) [10] and obesity (49%) [9] than those in the present study (100% and 71%, respectively). Thus, many participants in the PARAGON-HF trial may not have had NP deficiency syndrome, which could explain why the prognostic effect of Sac/Val was not more pronounced. Our study was planned and initiated before the pivotal PARAGON-HF trial results were published, which, in our opinion, enhances the value of our findings.

In the present study, treatment with Sac/Val was found to enhance functional capacity, as measured by the 6MWTD and bicycle exercise duration, over 6 months. This is clinically important because most HFpEF patients are elderly with severe comorbidities, putting them at high health risk [24]. Yet, the PARALLAX (Prospective Comparison of ARNI vs. Comorbidity-Associated Conventional Therapy on QOL and Exercise Capacity) trial, which enrolled 2572 patients with symptomatic HF and EF > 40%, found that Sac/Val did not improve 6MWTD over 24 weeks compared with standard RAAS inhibitors or placebo [25]. Again, many participants in the PARALLAX trial did not actually have NPs deficiency, as only half were obese, and many lacked LVH. Also, PARALLAX patients were older (mean age: 72.6 vs. 65.6 years), relatively frail (during the 6MWTD, they covered an average distance of only 305 m vs. 361 m), and had considerable comorbidities. Therefore, HF syndrome may not have been the main limiting factor in exercise tolerance.

In patients with obesity-associated HFpEF, poor exercise tolerance is mainly due to an excessive increase in LV filling pressure, driven by increased myocardial stiffness and plasma volume expansion [5]. In the present study, treatment with Sac/Val resulted in a reduction in LV filling pressure, as evidenced by a significant decrease in the E/e′ ratio, a primary indicator of diastolic function, both at rest and during exercise, as well as other echocardiographic parameters associated with LV filling pressure, including the LA volume index, E/SR_IVR_ ratio, PASP, and LASr. Our findings are in line with those of other studies. In the PARADISE-MI (Prospective ARNI Versus ACE Inhibitor Trial to Determine Superiority in Reducing Heart Failure Events After Myocardial Infarction) Echo Study, treatment with Sac/Val compared with ACEI ramipril after acute myocardial infarction resulted in greater improvement in LV filling pressure [26]. A meta-analysis of 8 studies in HF patients (mostly with low EF) revealed that Sac/Val significantly reduces the E/e′ ratio and LA volume index compared with standard therapies, with mean differences of −1.38 and −4.62, respectively (both *p* < 0.01) [27].

In the PARAMOUNT (Prospective Comparison of ARNI with ARB in the Management of Heart Failure with Preserved Ejection Fraction) trial, which included 301 patients with symptomatic heart failure and an ejection fraction (EF) of ≥45%, Sac/Val reduced LA volume to a greater extent than valsartan [28]. However, unlike the present study, which reported significant improvements in LV mass and echocardiographic diastolic parameters, the PARAMOUNT trial showed no differences in LV mass or diastolic function between treatment groups. Such differences between studies may suggest that the hemodynamic and structural effects of Sac/Val depend on the presence and severity of NP deficiency syndrome. Many participants in the PARAMOUNT trial did not have concentric LV hypertrophy (the mean LV mass index was only 77.5 g/m^2^ vs. 125 g/m^2^ in the present study) and were not overweight or obese (body mass index was 30.1 kg/m^2^ vs. 32.3 kg/m^2^ in the present study), and, consequently, did not have NP deficiency syndrome.

Both groups showed a comparable decrease in blood pressure (although 23% of patients in the Sac/Val group and none in the usual care group had asymptomatic arterial hypotension), and the degree of blood pressure reduction did not correlate with the potential treatment effect. We found that the effect of Sac/Val on LV filling pressure was dose-dependent, with the target dose (97/103 mg twice daily) providing the greatest improvement in LV filling pressure-associated parameters. In an open-label study of Sac/Val therapy in 727 HFrEF outpatients over 12.3 months, the incidence rate of the composite endpoint of death and hospitalization due to HF decreased progressively as Sac/Val dosage increased [29].

We were able to identify several Sac/Val-related effects that have the potential to enhance the mechanical properties of the myocardium. These include the regression of hypertrophy (a decrease in LV mass) and fibrosis (a decrease in blood levels of fibrotic biomarkers), and improved LV relaxation (an increase in SR_IVR_). Myocardial stiffness may be a major target for therapeutic intervention in patients with NP deficiency syndrome, since it worsens with both increasing body weight [30] and the severity of hypertrophy [31]. Furthermore, obesity significantly contributes to the development of LVH irrespective of blood pressure [32].

Since LV wall thickness is inversely proportional to diastolic stress and, accordingly, BNP production, the severity of concentric LVH can serve as a surrogate measure of NPs syndrome deficiency. Consequently, regression of LVH during antihypertensive treatment may indicate a favorable shift in the balance of forces governing NPs’ bioavailability. While data on LVH in HFpEF patients are limited, our study found that Sac/Val significantly reduced LV mass in HFpEF patients with concentric LVH. This may be accomplished by reducing afterload [33,34], as well as by suppressing the prohypertrophic/profibrotic angiotensin II signaling pathways and increasing the activity of the antihypertrophic/antifibrotic cGMP-PKG signaling pathways [7]. Schmieder et al. used magnetic resonance imaging to demonstrate a greater reduction in LV mass index with Sac/Val therapy compared to ARB olmesartan in patients with AH [35]. In their study, however, the Sac/Val group experienced a smaller reduction in LV mass index than in our study (≈−7 g/m^2^ vs. ≈−12 g/m^2^, respectively). In addition to discrepancies in LV mass assessment across imaging modalities, these differences may be attributed to the finding that a significant proportion of participants in Schmieder’s study did not exhibit LVH. In contrast, in the present study, many subjects exhibited advanced LVH, raising the concern of irreversible myocardial changes that could limit the efficacy of Sac/Val. However, further analysis revealed that the positive effects of Sac/Val were not influenced by baseline LVH severity. In the PARAGLIDE-HF (Prospective comparison of ARNI with ARB Given following stabiLization In DEcompensated HFpEF) study, the beneficial effect of Sac/Val on NT-proBNP and other efficacy endpoints in patients with LVEF > 40% and recent worsening HF also did not depend on LVH status [36]. Interestingly, the largest endomyocardial biopsy study of HFpEF to date found that, although histological signs of myocardial hypertrophy and fibrosis were present in most patients, only a minority had moderate or severe histopathological changes [37]. This has the potential to enhance the likelihood of reversal in both processes.

In this study, there were no significant changes in resting e′ velocity—a standard marker of relaxation—in the Sac/Val group. In addition to relaxation rates, e′ velocity depends on LV preload, i.e., the early diastolic lengthening force [38]. Since Sac/Val lowered preload (E velocity), likely, the opposing forces (the increasing force from improved relaxation and the decreasing force from reduced preload) balanced each other. Several reasons support this view. First, e′ velocity at rest fell in the usual care group but not in the Sac/Val group, with similar drops in preload. Second, the Sac/Val group improved in SR_IVR_, another relaxation marker [16]. Unlike e′ velocity, SR_IVR_ is independent of preload because it is measured during isovolumic relaxation when the mitral and aortic valves are closed.

Inhibiting neprilysin increases the activity of the intracellular cGMP-PKG signaling axis, which leads to the suppression of intracellular prohypertrophic signals, modulation of intracellular calcium transport, and myofilament phosphorylation [39]. The ability of Sac/Val to improve LV diastolic function has been demonstrated in several HFpEF murine models [40,41,42]. In these preclinical studies, Sac/Val also attenuated myocardial infiltration of inflammatory cells and fibrosis, thereby reducing LV passive stiffness. In the present study, Sac/Val treatment was associated with decreases in the pro-inflammatory marker hsCRP and the profibrotic marker osteopontin, consistent with other studies. In a study by Dhawan et al., Sac/Val demonstrated significant reductions in hsCRP when added to standard therapy in patients with HF [43]. In addition, in the PARAGON-HF trial, Sac/Val favorably altered biomarkers of extracellular matrix homeostasis compared with the ARB valsartan [44].

In patients with HFpEF, symptoms, particularly exertional dyspnoea, frequently mirror exercise hemodynamic abnormalities that may not be discernible at rest. In our study, Sac/Val therapy improved not only LV diastolic function at rest but also several cardiac reserves, including LV diastolic and preload reserves, ultimately leading to a decrease in LV filling pressure at peak exercise. The decrease in the peak E/e′ ratio occurred despite an increase in peak E velocity of 8% (peak LV inflow volume)—owing to an even greater (by 15%) increase in the peak e′ velocity—the denominator of the E/e′ ratio. From a mechanistic point of view, this means that, at peak stress, the LV was able to accommodate a greater blood volume with lower filling pressure. Such a hemodynamic effect of Sac/Val appears particularly relevant for obesity-related HFpEF, in which the interplay between elevated myocardial stiffness and plasma volume expansion results in a disproportionate increase in LV filling pressure at rest and during exercise [5]. To our knowledge, no prior studies have demonstrated improved cardiac reserve with Sac/Val therapy in patients with HFpEF.

Sac/Val has a well-established diuretic effect, which is expected to reduce LV preload. This may help address obesity-related plasma volume expansion, as was partly confirmed in our study by a reduction in E velocity at rest. In this regard, the improvement in preload reserve observed with Sac/Val (i.e., increased LV inflow during exercise) cannot be explained by fluctuations in blood volume. Rather, it appears to be due to improvements in RV contractile reserve, identified in the present study and shown to play an important role in improving LV filling during exercise [45]. This is confirmed by a significant correlation between changes in LV preload and RV contractile dynamics during Sac/Val therapy.

In patients with NP deficiency syndrome, NT-proBNP levels may be significantly lower than expected, even with elevated LV filling pressure [23]. Our patients had lower NT-proBNP levels than those in the PARAGON-HF trial (217 pg/mL vs. 904 pg/mL) [9]. However, the proportion of individuals with markers of increased LV filling pressure, such as LV enlargement (89%) and pulmonary hypertension (51%) in our cohort, was higher than in the PARAGON-HF trial (59% and 25%, respectively). In the context of NP deficiency syndrome, NT-proBNP remains to demonstrate its capacity to reflect LV filling pressure and its dynamics during treatment. The present study demonstrated significant correlations between the changes in NT-proBNP during Sac/Val therapy and the dynamics of echocardiographic parameters indicative of LV filling pressure.

On average, NT-proBNP levels decreased by 31% compared with those in the usual care group over 6 months. This observation was consistent with the results of the PARAGON-HF, PARAMOUNT, and PARAGLIDE-HF trials in patients with HF and mildly reduced/preserved EF, and elevated NT-proBNP levels, in which Sac/Val reduced NT-proBNP by 23%, 19%, and 15%, respectively, compared with valsartan (*p* < 0.05 for all) [28,46,47]. In the PARAGON-HF trial, the treatment effect of Sac/Val was found to be independent of the baseline NT-proBNP level. However, patients who exhibited the most significant reduction in NT-proBNP subsequently experienced the most favorable outcomes [46]. Notably, in our study, a decrease in NT-proBNP was observed in a cohort of patients with NP deficiency syndrome—that is, patients with normal or slightly elevated NT-proBNP levels (one-quarter had levels < 125 pg/mL)—and, consequently, there was little room for further reduction.

Among the limitations of the current study are the absence of a placebo control, the single-center design, the non-blinded design, and the relatively small number of participants. When assessing LV filling pressure, we relied on echocardiographic parameters, each of which has only a moderate association with invasively measured LV filling pressure [48]. Nevertheless, the consistent and significant improvement in all these markers following Sac/Val therapy indicates that Sac/Val reduces LV filling pressure in HFpEF patients with NP deficiency syndrome. We used 6MWTD and supine cycle ergometry (as part of the diastolic stress test) to assess exercise capacity. However, peak oxygen uptake (VO_2 peak_) during the cardiopulmonary exercise test is the most objective measure of subjects’ functional capacity. Nevertheless, 6MWTD and supine cycle ergometry yielded consistent beneficial results in the Sac/Val group, which, combined with the apparent improvement in echocardiographic parameters related to LV filling pressure, suggests that Sac/Val can improve functional capacity in our HFpEF cohort. In the present study, we included only patients with concentric LVH, whereas a significant proportion of patients with HFpEF have eccentric hypertrophy [10]. Nevertheless, concentric remodeling/LVH are considered the predominant patterns of structural LV abnormalities in HFpEF and are consistent with NP deficiency syndrome [49].

## 5. Conclusions

In this prospective, randomized study, treatment with Sac/Val improves functional capacity, along with favorable effects on LV mass and filling pressure (both at rest and at peak exercise) and biomarkers of inflammation and fibrosis in overweight or obese patients with HFpEF and concentric LVH. Our data therefore suggest that this patient cohort, who are thought to have NP deficiency syndrome, may benefit from Sac/Val therapy. However, due to the limitations of the current study, the efficacy of Sac/Val must be confirmed in larger randomized trials involving carefully selected patients who represent this common HFpEF phenotype.

## Figures and Tables

**Table 1:** Baseline characteristics of patients with HFpEF.

Variables	Total HFpEF Group (*n* = 61)	Sac/Val Group (*n* = 30)	Usual Care Group (*n* = 31)	*p* Value vs. Sac/Val
*Clinical parameters*	data	data		
Age, y	65.6 ± 8.5	66.0 ± 8.7	65.2 ± 8.5	0.71
Women, *n* (%)	35 (57)	17 (57)	18 (58)	0.91
NYHA II/III, *n* (%)	41/20 (67/33)	22/8 (73/27)	19/12 (61/39)	0.32
6-min walk test distance, m	361 ± 69	369 ± 81	351 ± 54	0.31
Arterial hypertension, ^a^ *n* (%)	61 (100)	30 (100)	31 (100)	1.0
Paroxysmal atrial fibrillation, *n* (%)	24 (40)	12 (40)	12 (39)	0.92
Ischemic heart disease, *n* (%)	19 (31)	11 (37)	8 (26)	0.36
Previous myocardial infarction, *n* (%)	5 (8)	3 (10)	2 (7)	0.64
Myocardial revascularization, *n* (%)	14 (23)	8 (27)	6 (19)	0.50
2 type diabetes mellitus, *n* (%)	21 (34)	11 (37)	10 (32)	0.72
HbA1c, % *	7.1 (6.4–7.8)	7.0 (6.2–7.7)	7.2 (6.5–7.9)	0.65
Body mass index, kg/m^2^	32.3 (29.4–36.5)	32.4 (29.6–36.2)	32.2 (28.7–36.5)	0.67
Overweight, ^b^ *n* (%)	18 (29)	8 (27)	10 (32)	0.64
Obesity, ^c^ *n* (%)	43 (71)	22 (73)	21 (68)	0.64
Severity of obesity:				0.99
Stage I (BMI 30.0–34.9 kg/m^2^)	16 (26)	8 (26)	8 (26)	16 (26)
Stage II (BMI 35.0–39.9 kg/m^2^)	21 (35)	11 (37)	10 (32)	21 (35)
Stage III (BMI ≥ 40 kg/m^2^)	6 (10)	3 (10)	3 (10)	6 (10)
Estimated GFR, mL/min/1.73 m^2^	68 ± 15	69 ± 14	68 ± 16	0.81
Chronic kidney disease, ^d^ *n* (%)	24 (39)	11 (37)	13 (42)	0.68
Systolic BP, mm Hg	140 (130–141)	140 (130–145)	138 (130–140)	0.82
Diastolic BP, mm Hg	90 (80–90)	90 (80–90)	90 (80–90)	0.63
Heart rate, bpm	65 ± 8	64 ± 6	67 ± 10	0.23
NT-proBNP, pg/mL	217 (125–315)	226 (148–359)	203 (92–306)	0.51
NT-proBNP level < 125 pg/mL, *n* (%)	15 (25)	7 (23)	8 (26)	0.82
*Baseline treatments*:				
ACEI/ARB (prestudy), *n* (%)	61 (100)	30 (100)	31 (100)	1.0
β-Blockers, *n* (%)	38 (63)	21 (70)	17 (55)	0.23
Loop diuretics, *n* (%)	45 (74)	23 (77)	22 (71)	0.62
MRA, *n* (%)	18 (30)	10 (33)	8 (26)	0.52
Calcium channel blockers, *n* (%)	40 (66)	20 (67)	20 (65)	0.86
Statins, *n* (%)	54 (89)	28 (93)	26 (84)	0.25
SGLT2 inhibitors, *n* (%)	16 (26)	8 (27)	8 (26)	0.94
Glucagon-like peptide-1 agonists, *n* (%)	4 (7)	2 (7)	2 (7)	0.97
*Echocardiographic measures*				
LV ejection fraction, %	61.6 ± 6.0	61.4 ± 6.6	61.8 ± 5.5	0.79
LV mass index, g/m^2^	124 (114–141)	125 (115–142)	123 (114–137)	0.40
LV hypertrophy, ^e^ *n* (%) Mild, *n* (%) Moderate, *n* (%) Severe, *n* (%)	61 (100) 21 (34) 19 (31) 21 (34)	30 (100) 11 (37) 8 (26) 11 (37)	31 (100) 10 (32) 11 (36) 10 (32)	
LA volume index, mL/m^2^	42.3 (36.9–49.3)	43.5 (39.5–49.6)	40.6 (35.7–49.1)	0.29
LA dilatation, ^f^ *n* (%)	54 (89)	28 (93)	26 (84)	0.25
LASr, %	22.3 ± 5.3	21.3 ± 5.5	23.3 ± 5.1	0.15
LA dysfunction, ^g^ *n* (%)	39 (64)	22 (73)	17 (55)	0.14
E/e′ ratio	14.4 (11.5–17.0)	14.3 (11.0–17.1)	14.8 (12.2–16.9)	0.48
LV DD grade II-III, *n* (%)	43 (70)	22 (73)	21 (68)	0.78
Estimated PASP, mm Hg	35.8 (30.1–40.7)	36.9 (32.8–40.7)	34.8 (28.7–40.4)	0.14
Pulmonary hypertension, ^h^ *n* (%)	31 (51)	17 (57)	14 (45)	

Data are presented as the means ± standard deviations for continuous normally distributed variables, medians (25th–75th percentiles) for nonnormally distributed continuous variables, and frequencies (%) for categorical variables. *—among patients with diabetes; ^a^—blood pressure ≥ 140/90 Hg mm; ^b^—body mass index ≥ 25 kg/m^2^; ^c^—body mass index ≥ 30 kg/m^2^; ^d^—eGFR < 60 mL/min/1.73 m^2^; ^e^—LV mass index > 115 g/m^2^ in men and >95 g/m^2^ in women; mild LVH: LV mass index 116–131 g/m^2^ in men and 96–108 g/m^2^ in women, moderate LVH: LV mass index 132–148 g/m^2^ in men and 109–121 g/m^2^ in women, severe LVH: LV mass index > 148 g/m^2^ in men and >121 g/m^2^ in women; ^f^—LA volume index > 34 mL/m^2^; ^g^—LASr < 23%; ^h^—pulmonary artery systolic pressure > 35 mm Hg. ACEI, angiotensin-converting enzyme inhibitor; ARB, angiotensin receptor blocker; BMI, body mass index; BP, blood pressure; DD, diastolic dysfunction; E, early inflow velocity; e′, averaged annulus relaxation velocity; GFR, glomerular filtration rate; HbA1c, glycosylated hemoglobin; HFpEF, heart failure with preserved ejection fraction; LA, left atrial; LASr, left atrial strain during reservoir phase; LV, left ventricular; MRA, Mineralocorticoid receptors antagonist; NYHA, New York Heart Association; NT-proBNP, N-terminal pro-brain natriuretic peptide; PASP, pulmonary artery systolic pressure; SGLT2, sodium-glucose transport protein 2.

**Table 2:** Dynamics of clinical and laboratory parameters, biomarker levels, and resting echocardiographic measures in the study groups during the follow-up period.

Variables	Sac/Val Group (*n* = 30)		Usual Care Group (*n* = 31)		Difference Between Groups (95% CI)	*p*-Value for Changes’ Comparison
	Baseline	Δ from Baseline (95% CI)	Baseline	Δ from Baseline (95% CI)		
*Clinical parameters*						
Systolic BP, mm Hg	138 (128–146)	−6 (−12, −1)	138 (132–142)	−3 (−5, 0.1)	2 (−2, 10)	0.30
Diastolic BP, mm Hg Heart rate, bpm 6MWTD, m	88 (80–90)	−4 (−7, −1)	86 (79–90)	−2 (−4, 0)	2 (−1, 6)	0.18
	64 ± 6	0 (−3, 2)	67 ± 10	1 (−2, 4)	1 (−3, 5)	0.74
	369 ± 81	15 (8, 22)	351 ± 54	3 (−3, 10)	−12 (−21, −3)	0.010
Exercise duration, s	459 (344–600)	80 (44, 111)	423 (304–536)	22 (−16, 59)	−57 (−104, −11)	0.018
*Biochemistry measures and biomarkers*						
NT-proBNP, pg/mL	226 (148–359)	−81 (−143, −54)	203 (92–306)	1 (−15, 29)	81 (49, 135)	<0.0001
hsCRP, mg/L	1.7 (1.0–4.9)	−0.7 (−1.6, −0.4)	1.8 (0.9–3.1)	−0.1 (−0.3, 0.1)	0.6 (0.3, 1.1)	<0.001
Osteopontin, pg/mL	135 (82–241)	−19 (−50, −4)	143 (74–251)	9 (−11, 33)	−29 (−56, −1)	0.033
Creatinine, μmol/L	84 (79–105)	−2 (−5, 2)	89 (82–104)	1 (−2, 5)	3 (−1, 8)	0.11
*Echocardiographic measures*						
LV mass index, g/m^2^	125 (115–142)	−12 (−16, −7)	123 (115–137)	−4 (−7, 0)	8 (2, 13)	0.007
LV EDD, mm	47.9 ± 4.3	−1.2 (−2.2, −0.2)	47.5 ± 4.6	−1.0 (−1.9, −0.1)	0.2 (−1.1, 1.5)	0.78
LV EDV, mL	94 (79–115)	−6 (−12, 1)	93 (79–118)	−1 (−5, 3)	2 (−2, 6)	0.28
LV ejection fraction, %	61.4 ± 6.6	0.8 (−1.0, 2.6)	61.8 ± 5.5	0.7 (−0.9, 2.3)	−0.1 (−2.5, 2.2)	0.91
LA volume index, mL/m^2^	43.5 (39.5–49.6)	−2.4 (−4.2, −0.7)	40.6 (35.7–49.1)	0.1 (−1.2, 1.8)	2.6 (0.2–4.7)	0.026
LASr, %	21.3 ± 5.5	1.7 (0.6, 2.8)	23.3 ± 5.1	−0.1 (−1.0, 0.8)	−1.8 (−3.2, −0.4)	0.012
SR_IVR_, s^–1^	0.26 (0.21–0.39)	0.08 (0.03, 0.14)	0.36 (0.19–0.45)	0.01 (−0.03, 0.05)	−0.07 (−0.13,−0.01)	0.019
E/SR_IVR_ ratio, cm	301 (223–400)	−110 (−166, −54)	295 (192–443)	−21 (−85, 33)	90 (19, 157)	0.023
E velocity, cm/s	83 (70–97)	−6 (−11, 0)	91 (75–108)	−6 (−11, −1)	−1 (−8, 6)	0.84
Annular e′ velocity, cm/s	5.9 (4.9–6.9)	0.0 (−0.5, 0.5)	6.3 (5.3–7.1)	−0.5 (−0.7, −0.2)	−0.6 (−1.1, 0.1)	0.075
E/e′ ratio	14.3 (11.0–17.1)	−1.1 (−2.0, −0.1)	14.8 (12.2–16.9)	−0.1 (−0.8, 0.7)	1.0 (0.02–2.3)	0.045
Estimated PASP, mm Hg	36.9 (32.8–40.7)	−4.9 (−7.6, −1.8)	34.8 (28.7–40.4)	−0.2 (−3.2, 2.9)	4.5 (0.5, 8.7)	0.024
TR velocity, m/s	2.66 (2.45–2.90)	−0.18 (−0.30, −0.05)	2.67 (2.55–2.83)	0.05 (−0.06, 0.16)	0.23 (0.07, 0.39)	0.007

Baseline data are presented as the mean ± standard deviation for normally distributed continuous variables and the median (25th–75th percentile) for nonnormally distributed continuous variables; the dynamics of variables are presented as the mean change from baseline (95% confidence interval). EDD, end-diastolic dimension; EDV, end-diastolic volume; hsCRP, high-sensitivity C-reactive protein; SR_IVR_, global strain rate during isovolumic relaxation period; TR, tricuspid regurgitation; 6MWTD, 6-min walk test distance. Other abbreviations are as in Table 1.

**Table 3:** Dynamics of exercise echocardiographic measures in the study groups.

Variables	Sac/Val Group (*n* = 30)		Usual Care Group *(n* = 31)		*p*-Value for Intergroup Differences
	Baseline of the Study	6 Months of Study	Baseline of Study	6 Months of Study	
*E, cm/s*					
Rest Peak ∆ Rest-to-Peak ^a^	83 (70–97)	76 (60–91) *	91 (75–108)	86 (69–100) *	–
	140 (112–170)	152 (130–176) **	153 (137–182)	157 (130–175)	–
	57 (43 to 75)	76 (66 to 85) **	65 (58 to 76)	71 (54 to 83)	–
Changes in ∆ Rest-to-Peak		+19 (11 to 28)		−2 (−9 to 5)	0.009
*e* *′* *, cm/s*					
Rest	5.9 (4.9–6.9)	6.1 (5.3–7.0)	6.3 (5.3–7.1)	5.8 (4.8–6.7) **	–
Peak	8.6 (8.0–10.4)	9.8 (8.8–11.5) **	9.2 (7.8–10.4)	8.5 (7.2–10.4) ^§^	–
∆ Rest-to-Peak ^a^	2.9 (2.5 to 3.4)	4.1 (3.4 to 4.7)	3.0 (2.5 to 3.4)	3.1 (2.5 to 3.7)	–
Changes in ∆ Rest-to-Peak		+1.1 (0.4 to 1.8)		0.0 (−0.3 to 0.3)	0.010
*E/e* *′* *ratio*					
Rest	14.3 (11.0–17.1)	12.7 (10.8–14.4) *	14.8 (12.2–16.9)	13.3 (12.1–18.0)	–
Peak	16.8 (14.9–18.5)	14.9 (12.8–18.1) **	17.3 (14.5–19.9)	16.5 (14.5–21.0) ^§^	–
∆ Rest-to-Peak ^a^	3.2 (2.5 to 3.9)	2.2 (1.5 to 3.0)	2.9 (2.1 to 3.8)	3.0 (2.2 to 3.6)	–
Changes in ∆ Rest-to-Peak		−1.1 (−1.9 to 0.0)		0.1 (−0.9 to 1.0)	0.053
*TR velocity, m/s*					
Rest	2.66 (2.45–2.90)	2.51 (2.36–2.69) **	2.67 (2.55–2.83)	2.70 (2.51–2.93)	–
Peak	3.59 (3.35–3.86)	3.47 (3.21–3.75) **	3.55 (3.19–3.81)	3.60 (3.15–3.94)	–
∆ Rest-to-Peak ^a^	0.93 (0.79 to 1.08)	0.96 (0.81 to 1.10)	0.84 (0.70 to 0.97)	0.83 (0.73 to 0.95)	–
Changes in ∆ Rest-to-Peak	-	0.01 (−0.13 to 0.11)		0.00 (−0.14 to 0.15)	0.94
*TAPSE, cm*					
Rest	2.02 (1.85–2.17)	1.95 (1.80–2.18)	2.02 (1.84–2.19)	1.84 (1.67–2.11)	–
Peak	2.36 (2.15–2.62)	2.42 (2.21–2.71)	2.44 (2.17–2.70)	2.30 (2.08–2.55) ^§^	–
∆ Rest-to-Peak ^a^	0.36 (0.25 to 0.49)	0.51 (0.35 to 0.65)	0.41 (0.30 to 0.51)	0.38 (0.27 to 0.51)	–
Changes in ∆ Rest-to-Peak		+0.13 (0.00 to 0.29) *		0.00 (−0.11 to 0.11)	0.089

Baseline data at rest are presented as the mean ± standard deviation; the dynamics of variables and cardiac reserves are presented as the mean change from the baseline values (95% confidence interval). ^a^—the changes in all parameters from rest to peak exercise in both groups, at the initial visit and after 6 months, were highly significant (*p* < 0.001). * *p* < 0.05, ** *p* < 0.01 vs. baseline (intragroup differences); ^§^ *p* < 0.05 vs. atorvastatin group (intergroup differences). E, early inflow velocity; e′, averaged annulus relaxation velocity; TR, tricuspid regurgitation.

## Data Availability

Data used in this study are available from the corresponding author upon reasonable request.

## Abbreviations

The following abbreviations are used in this manuscript:

ACE	Angiotensin-converting enzyme
AcT	Acceleration time
AH	Arterial hypertension
ARB	Angiotensin receptor blocker
cGMP	Cyclic guanosine monophosphate
CI	Confidence interval
CAD	Coronary artery disease
CKD	Chronic kidney disease
DM	Type 2 diabetes mellitus
DST	Diastolic stress test
E	Early inflow velocity
e′	Annulus relaxation velocity
EF	Ejection fraction
HFpEF	Heart failure with preserved ejection fraction
hsCRP	High-sensitivity C-reactive protein
LA	Left atrial
LASr	Left atrial strain during the reservoir phase
LAVI	Left atrial volume index
LV	Left ventricular
DD	Diastolic dysfunction
LVH	Left ventricular hypertrophy
LVMI	Left ventricular mass index
NP	Natriuretic peptide
NT-proBNP	N-terminal pro-brain natriuretic peptide
NYHA	New York Heart Association
PASP	Pulmonary artery systolic pressure
PKG	Protein kinase G
RA	Right atrial
RAAS	Renin–angiotensin–aldosterone system
RV	Right ventricular
RVOT	Right ventricular outflow tract
RWT	Relative wall thickness
Sac/Val	Sacubitril/valsartan
SR_IVR_	Strain rate during isovolumic relaxation
TAPSE	Tricuspid annular plane systolic excursion
TR	Tricuspid regurgitation
6MWTD	Six-minute walk test distance
